# Thigh Pain and Peri-Implant Fractures with the Use of Short Cephalo-medullary Nails: A Retrospective Study of 122 Patients

**DOI:** 10.5704/MOJ.2211.004

**Published:** 2022-11

**Authors:** S Dubey, RD Iyer, MQ Azam, B Sarkar, H Nongdamba

**Affiliations:** 1 Department of Orthopaedics, Shri Ram Murti Smarak Institute of Medical Sciences, Bareilly, India; 2 Department of Orthopaedics, All India Institute of Medical Sciences, Raipur, India; 3 Department of Trauma Surgery, All India Institute of Medical Sciences, Rishikesh, India; 4 Department of Orthopaedics, All India Institute of Medical Sciences, Rishikesh, India

**Keywords:** intertrochanteric fractures, short nail, thigh pain, peri-implant fractures, re-fractures

## Abstract

**Introduction:**

To assess the incidence and causes of persistent thigh pain and peri-implant fractures after union in patients of intertrochanteric fractures treated with short cephalo-medullary nails.

**Materials and methods:**

A retrospective observational study conducted at a Level 1 Trauma centre. A total of 122 patients of intertrochanteric fractures who were operated using short cephalo-medullary nails (170mm and 200mm lengths) between January 2018 to June 2019 were included in the study. Main outcomes measured were the incidence of thigh pain and peri-implant fractures.

**Results:**

Out of the 122 patients with a mean follow-up of 14.1 month, 12 patients had persistent thigh pain. Six patients had the helical blade protruding from the lateral cortex, two of them had distal tip of nail abutting on the anterior cortex and four cases had prominent proximal segment of nail which may explain the cause of their pain. Five of these patients had a combination of these findings. Two patients had pain for which no other obvious cause was found. There were no cases of peri-implant fractures in our study.

**Conclusion:**

Thigh pain associated with the use of short cephalon-medullary nails is often unrelated to nail length and can be prevented by using proper surgical technique. There seems to be no association between the use of short nails and peri-implant fractures.

## Introduction

Fractures around hip are debilitating injuries. They are associated with high mortality rates, reported to be around 10% in some studies as well as morbidity associated with immobilisation in elderly patients^1,2^. Management has evolved from conservative management to early surgical intervention wherever feasible, to allow early mobilisation. Most of the older literature suggests the use of long proximal femoral nails for intertrochanteric fractures in elderly osteoporotic patients, the rationale being splintage of entire femur using a long nail reduces the chances of peri-implant fractures and also negates the chances of thigh pain due to impingement of nail tip against the anterior femoral cortex^3^. However, using long nails for all such cases is fraught with its own problems and risks. Use of short nails have numerous advantages like shorter operative and anaesthesia time, less blood loss, less reaming (reduced chances of emboli) and reduced chances of distal perforation, all of which have been proven in numerous studies^4-7^. In spite of all these advantages there is a theoretically increased risk of peri-implant failures and thigh pain with the short nails. Clinical results of short nails are comparable to their longer counterparts even with theoretical biomechanical handicap. Many surgeons have also begun to use distally unlocked short nails in intertrochanteric fractures with good clinical outcomes^8,9^.

There are far too few studies focussing on the aspect of thigh pain and re-fractures after surgery because of which there is huge void in our knowledge about the incidence, aetiology, and ways to prevent these complications associated with short nails. Also, there are many conflicting views about the aetiology of pain in these patients which cannot be attributable to the nail size alone.

In this study we have tried to find the answer to the question that whether there is any significant increase in the incidence of thigh pain and peri-implant fractures in patients of intertrochanteric fractures operated using short cephalomedullary nails. We have also tried to ponder about the probable causes of pain when present and whether it can be attributed to the size of the nail used.

## Materials and Methods

After institutional review board approval, a retrospective study was conducted on patients of intertrochanteric fractures operated at our institute with short cephalomedullary nails (170mm and 200mm) between January 2018 and June 2019. The aim of the study was to assess the incidence and causes of thigh pain and peri-implant fractures in these patients. All patients over 60 years of age with closed isolated intertrochanteric fractures (AO/OTA 31A1, 31A2 and 31A3)^10^ managed operatively using short cephalomedullary nail with clinical and radiographic signs of union with a minimum follow-up of 12 months were included in the study. We excluded patients of polytrauma, pathological fractures, revision surgery, non-union and implant failure.

**Fig. 1.**
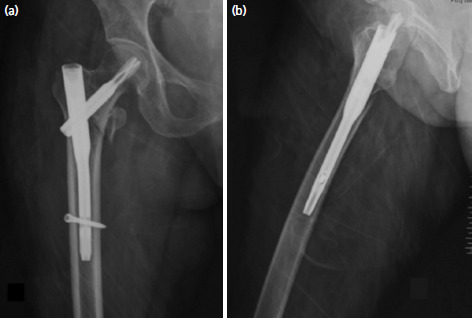
(a) Antero-posterior (AP) radiograph showing good reduction and implant positioning using short cephalo-medullary nail, (b) lateral radiograph of the same patient.

After adequate effect of anaesthesia, patient was positioned on the traction table and closed reduction attempted. After successful closed reduction, internal fixation was done using standard technique of cephalo-medullary nailing with short nail with helical blade [PROFAN, Nebula Surgicals] (Fig. 1). Post-operative protocol was uniform for all patients. Toe-touch weight bearing was allowed from post op day one. Complete weight bearing was allowed after radiographic union. Patients were followed at 2 weeks, 6 weeks, 12 weeks, 6 months, 12 months and yearly thereafter.

Medical records were studied for obtaining the demographic data, surgical details, radiographs, and information regarding the implant. Outpatient records were studied to retrieve data regarding follow-up. SPSS v23 [IBM corp.] was used for data analysis. At each follow-up visits the patients were enquired regarding thigh pain. Intensity of thigh pain when present was quantified using visual analogue scale (VAS). If they complained of thigh pain, we further enquired about the site of pain, onset of pain (since post-operative period or after some time lapse), nature of pain and its relationship to ambulation (whether pain is relieved on rest or not). Mobility status of the patients were quantified using the modified Harris hip score (mHHS)^11^ and was compared it to its pre-trauma status. Descriptive statistics were elaborated in form of mean/standard deviations for continuous variables and frequency/percentage for categorical variable. Group comparisons for continuously distributed data were made using independent 't' test. Chi-squared test was used for group comparisons of categorical data. In cases where the expected frequency in contingency table was less than 5 for >25% cells, Fischer's exact test was used instead. Statistical significance was kept at p value <0.05.

## Results

A total of 122 patients of intertrochanteric fractures satisfying our inclusion criteria was found in our records. There were 79 males and 43 females. The mean (SD) age of patients included in the study was 70.18 (6.94) years. The basic demographic data of our patients is showed in Table I. Twelve patients complained of persistent thigh pain at last follow-up. Association between thigh pain and various parameters are summarised in Table II. The variable age (years) was not normally distributed in the two subgroups of the variable thigh pain. Thus, non-parametric tests (Wilcoxon-Mann-Whitney U Test) were used to make group comparisons.

**Table I:** Patient demographic data.

Number of patients	122
Mean age (range)	70.18 years (60 - 89 years)
Male/Female ratio	1.77:1 (Males-79, Females-43)
Mean follow up period	14.1 months (12 - 18 months follow-up)
Mean pre-trauma mobility score (using modified Harris hip score)	88.6
Mode of injury	High velocity trauma - 26
	Low velocity trauma - 96
Size of nail used	170mm - 67
	200mm - 55

*Independent sample t-test

**Table II:** Association between thigh pain and other variables.

Parameters	Thigh Pain Present (n = 12)	Absent (n = 110)	p value
Age (Years)	70.67 ± 4.66	70.13 ± 7.16	0.651^1^
Gender			0.434^2^
Male	9 (75.0%)	70 (63.6%)	
Female	3 (25.0%)	40 (36.4%)	
Nail Length			1.000^3^
170mm	7 (58.3%)	60 (54.5%)	
200mm	5 (41.7%)	50 (45.5%)	

***Significant at p<0.05, 1: Wilcoxon-Mann-Whitney U Test, 2: Fisher's Exact Test, 3: Chi-Squared Test

The mean (SD) of Age (Years) in the group with thigh pain was 70.67 (4.66) with an age range of 64 - 78 years. The mean (SD) of Age (Years) in the group without thigh pain was 70.13 (7.16) with the age range of 60 - 89. There was no significant difference between the groups in terms of Age (Years) (W = 713.000, p = 0.651). Mean time for radiographic union was 20.2 weeks.

Fisher's exact test was used to explore the association between 'Thigh Pain' and 'Gender' as more than 20% of the total number of cells had an expected count of less than five. There was no significant difference between the various groups in terms of distribution of Gender (χ2 = 0.612, p = 0.434).

Twelve patients (9.8%) complained of thigh pain at follow-up. Out of the twelve patients eight of them independently mobile while four patients needed cane/stick. None of the patients had peri-implant fractures, even though five patients had history of fall at home after surgery. The mean pain score using visual analogue scale was 3.25 with individual scores ranging between 2-4. The pain was mild to moderate in intensity and did not affect their mobility or ability to perform activities of daily living with an average modified Harris hip score^11^ of 82.5. The pain was localised to proximal thigh in all the patients and not associated with groin pain or tingling or paraesthesia ruling out any other causes like meralgia paraesthetica, co-existing hip arthritis, blade cut through which was confirmed by evaluation of their radiographs.

Six patients had prominent proximal blade due to improper length of the helical blade. Five of them had point of maximum tenderness over the proximal blade insertion site while others had diffuse pain over proximal thigh. However, only two of them had long blade as the only radiographic finding while the rest had some other associated findings.

Two cases had too posterior an entry point which led to nail abutment at the anterior cortex while three cases had excessive bowing of femur that led to similar finding. Four cases had a prominent proximal nail segment, out of which in only one patient was it the only associated finding. Five of the twelve patients had one of the above causes as isolated finding. The other five had a combination of findings as summarised in Table III. Two patients did not have any other radiographic finding which may explain the thigh pain. Eight patients had shortening of limb although the limb length discrepancy (LLD) was less than 10mm. None of the patients had thigh pain. Chi-squared test was used to explore the association between 'Thigh Pain' and 'Nail Length'. There was no significant difference between the 170m and 200mm groups (χ^2^ = 0.063, p = 1.000).

**Table III:** Details of patient with thigh pain at follow-up.

S No.	Age	Sex	Nail Size	Pain score (using Visual analogue scale)	Associated radiological findings	Activity level and mHHS at follow-up
1.	64 yrs	M	170mm	3	Long blade	77
2.	70 yrs	F	170mm	2	Long blade, Proud nail	77
3.	70 yrs	M	170mm	3	Long blade, Proud nail	83
4.	67 yrs	M	170mm	3	Posterior entry point	87
5.	75 yrs	M	170mm	4	Long blade	81
6.	78 yrs	M	170mm	3	Long blade, Excessive bowing of femur	87
7.	72 yrs	F	170mm	3	Excessive bowing of femur, Proud nail	77
8.	65 yrs	M	200mm	3	No specific finding	87
9.	72 yrs	F	200mm	4	Long blade, posterior entry	81
10.	65 yrs	F	200mm	4	Proud nail	83
11.	74 yrs	M	200mm	3	No specific finding	87
12.	76 yrs	M	200mm	4	Excessive bowing of femur	83
**Associated radiographic findings**						**Number of cases**
Improper blade length (Lateral soft tissue irritation)						6
Proud nail segment proximally						4
Excessive bowing of femur						3
Posterior entry point						2
Multiple factors						5
No radiographic finding						2

## Discussion

Thigh pain and peri-implant fractures has been reported as drawbacks of using short nails. Advantages offered by short nails are alluring but at the same time apprehension of these pitfalls often creates a dilemma in the mind of treating surgeon regarding ideal choice of implants. Hence, we conducted this study to assess the actual magnitude of these impediments while using short CMN.

**Fig. 2.**
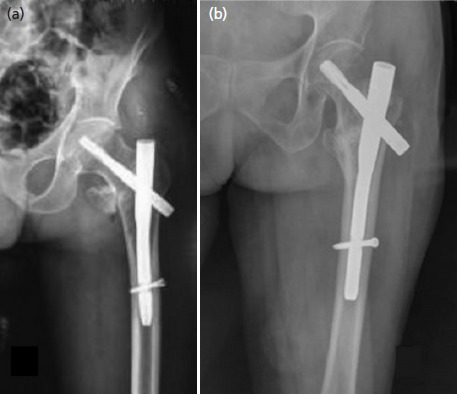
(a, b) Antero-posterior radiographs of two different patients showing union at the fracture site with protrusion of helical blade beyond the lateral cortex. At follow-up both patients still complain of thigh pain with tenderness over the blade insertion site.

**Fig. 3.**
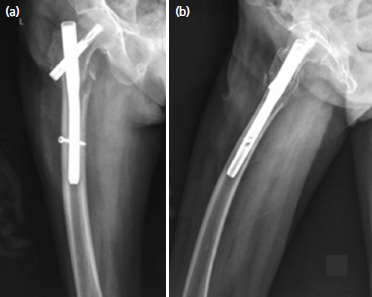
(a) Radiograph showing excessive bowing of femur, which is not uncommon in our elderly population. (b) Lateral radiograph of the same patient with excessive curvature in the sagittal plane as well.

**Fig. 4.**
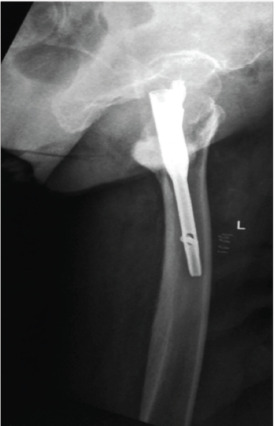
Radiograph showing anterior cortical abutment of nail tip due to excessively posterior entry point.

In our study, we observed that the short versions of the cephalo-medullary nail (170mm and 200mm) was associated with thigh pain in 12 patients (9.8%). We did not encounter any cases of peri-implant fractures even though five patients reported low energy trauma (fall at home) after surgery. We further examined and analysed the radiographs of each patient who complained of thigh pain at follow-up. We found that majority of the cases had a finding (clinical or radiological) which can explain the reason for thigh pain apart from the nail length. The most common findings were improper length of helical blade with lateral overhang of the blade, causing irritation of the vastus lateralis and tensor fascia lata muscles (Fig. 2). This was confirmed clinically by localising the point of maximum tenderness around the blade insertion site which was relieved by infiltration of local anaesthetic agent around that site. Other common causes encountered were long proximal segment above the greater trochanter, excessive bowing of femur (Fig. 3) and too posterior entry point causing anterior cortex abutment (Fig. 4). In two cases of thigh pain no other attributable cause could be found. Shortening due to fracture impaction is frequently encountered with the use of sliding hip screws, though not uncommon with the use of CMNs. Significant limb length discrepancy (LLD greater than 2.5cm) if present are an important cause of limping and hip pain and must be evaluated. In our study we did not encounter any cases of significant LLDs are usually encountered with varus collapse and non-union cases which we had excluded. Based on these observations, we opine that most of the “probable causes” of thigh pain are preventable by using proper surgical technique and implant selection which would not be alleviated by the use of a longer version of nail.

There is a lacuna in our current understanding on the subject of thigh pain and peri-implant fractures after proximal femoral nailing due to lack of objective data and quality evidence. A study by Dodenhoff *et al*^12^ on proximal thigh pain after femoral nailing with Grosse-Kempf nail for diaphyseal fractures observed that heterotrophic ossification was a major cause of persistent thigh pain. However, we did not encounter any case of heterotrophic ossification in our study. The study also sheds light on other probable causes, but the underlying point being that thigh pain is also associated with long nail and short nail length may not be a risk factor as has been the common notion.

A study by Jin-Song Pu *et al*^13^ highlighted the problem of mismatch of nail and femur geometry. In their study they encountered problems related to the mismatch of the proximal end of the nail in 11 cases. Nine of these patients presented with thigh pain due to the redundant proximal end of the nail. In another study by Rosen *et al*^14^, they commented that lateral hip pain from proximal locking device insertion and prominence continues to be one of the most frequent complaints following this surgical procedure. Another factor for thigh pain is the length of the nail chosen. There is no Level I or II evidence that favour one nail length over other (170mm, 200mm or 220mm). In a randomised controlled trial conducted by Parker and Cawley^15^ they concluded that there was a trend toward greater residual pain for those treated with the shorter nail, although this was not statistically significant and cautioned about the excessive use of these short versions of the nail. Though there is no biomechanical study that has determined the ideal length of nail for cephalo-medullary nailing, there has been a trend of decreasing size of the implants from 220-250mm nails to the shorter versions (170-200mm nails). Nails as short as 160mm have been used in some situations with good clinical results^16^. In this study we did not find any significant difference in the incidence of peri-implant fractures or thigh pain between 170mm and 200mm nails.

In a study by Muller *et al*^17^, they concluded that though peri-implant fractures were rare incidences, chances of its occurrence were 3.7 times higher with PFN compared to DHS. Numerous studies have attempted to evaluate these factors but have their own set of flaws, like use of previous generations of nails (gamma nails were used in most of the studies), longer length of nails (240-250mm nails were more commonly used in the past) and nail-femur geometry mismatch. In a study by Robinson *et al*^18^, they found that the risk of femoral peri-implant fracture after hip surgery was 40 times more than general population. However, these previous generations of short nails used, had many flaws. They were rigid, stainless-steel implants with large locking bolts at the distal tip of the nail in the diaphyseal region. Also, there was frequent mismatch of the nail and femoral geometry that frequently created stress risers. In addition, these original relatively large diameter short nails were associated with intra-operative insertional fractures. The problems have largely been addressed in the newer generations of nail which have taper stem, oblique distal bolts and more anatomical bend leading to lesser incidence of peri-implant fractures.

Another important and upcoming problem is the exponential increase in total knee arthroplasties. With the increased in longevity, more and more patients suffering from knee osteoarthritis are opting for arthroplasty. Use of stem in femoral component would not be possible in presence of long nail and hence we need to consider short nails in these patient populations. This makes it even more important to understand in detail, the pros and cons associated with the use of short nails in geriatric population.

The lifestyle and mobility of elderly population in Asian countries differs significantly from the western populations. Most of the elderly population, even before the trauma are confined mostly to low demand home-bound activities like stair climbing, morning walks and kneeling for prayers. In contrast, the elderly in western countries lead a more demanding lifestyle including independent driving, routine exercises and in some cases participation in active sports. This may explain the lower incidence of peri-implant fractures and thigh pain in our study compared to other studies involving western populations. However, this demographic and lifestyle differences may change when the current generations born after 1990s ages as their lifestyle and mobility expectations are quite similar to the western populations. It would be interesting to see if the short nails survive this test of time.

The limitations of the study, firstly, relatively small sample size, which may affect the quality of final outcome. Another limitation is the short duration of follow-up (mean follow-up of 14.1 months). Though we believe that more than 12 months of follow-up is sufficient for peri-trochanteric fractures for union and clinical outcomes, the incidence of peri-implant fractures and thigh pain need longer periods of follow-up. We plan to continue the study on our patients and document the long-term results with regard to the parameters considered in this study.

## Conclusion

Thigh pain associated with the use of short cephalomedullary nails is often unrelated to nail length and can be prevented by using proper surgical technique. There is no association between the use of short nails and peri-implant fractures. Modern short nail designs are much safer with respect to peri-implant fractures as evident by no cases in our study. However, further better designed clinical studies with larger sample size and longer follow-up are required to make the evidence unequivocal.
